# Barriers faced by patients in the diagnosis of multidrug-resistant tuberculosis in Brazil

**DOI:** 10.11606/s1518-8787.2022056004154

**Published:** 2022-06-20

**Authors:** Marcela Bhering, Margareth Dalcolmo, Vicente Sarubbi, Afrânio Kritski

**Affiliations:** I Fundação Oswaldo Cruz Escola Nacional de Saúde Pública Sérgio Arouca Rio de Janeiro RJ Brasil Fundação Oswaldo Cruz. Escola Nacional de Saúde Pública Sérgio Arouca. Rio de Janeiro, RJ, Brasil; II Universidade Federal do Rio de Janeiro Faculdade de Medicina Programa Acadêmico de Tuberculose Rio de Janeiro RJ Brasil Universidade Federal do Rio de Janeiro. Faculdade de Medicina. Programa Acadêmico de Tuberculose. Rio de Janeiro, RJ, Brasil; III Universidade Estatual de Mato Grosso do Sul Faculdade de Medicina Campo Grande MS Brasil Universidade Estatual de Mato Grosso do Sul. Faculdade de Medicina. Campo Grande, MS, Brasil

**Keywords:** Patients, Tuberculosis, Multidrug-Resistant, diagnosis, Barriers to Access of Health Services, Health Knowledge, Attitudes, Practice, Qualitative Research

## Abstract

**OBJECTIVE:**

To understand patients’ narratives about the barriers they faced in the diagnosis and treatment of multidrug-resistant tuberculosis, and their consequences in Rio de Janeiro State, Brazil.

**METHODS:**

This is a qualitative cross-sectional study with non-probabilistic sampling. A theoretical saturation criterion was considered for composing the number of interviewees. Semi-structured interviews were conducted from August to December 2019 with 31 patients undergoing treatment for multidrug-resistant tuberculosis at an outpatient referral center in Rio de Janeiro. Data were transcribed and processed with the aid of the NVIVO software. Interviews were evaluated by content analysis, and their themes, cross-referenced with participants’ characterization data.

**RESULTS:**

Our main findings were: a) participants show a high proportion of primary drug resistance, b) patients experience delays in the diagnosis and effective treatment of multidrug-resistant tuberculosis ; c) healthcare providers fail to value or seek the diagnosis of drug-resistant tuberculosis, thus beginning the inadequate treatment for drug-susceptible tuberculosis, d) primary health units show low report rates of active case-finding and contact monitoring, and e) patients show poor knowledge about the disease.

**CONCLUSIONS:**

We need to improve referral systems, and access to the diagnosis and effective treatment of multidrug-resistant tuberculosis; conduct an active investigation of contacts; intensify the training of healthcare providers, in collaboration with medical and nursing schools, in both public and private systems; and promote campaigns to educate the population on tuberculosis signs and symptoms.

## INTRODUCTION

Brazil appears on the World Health Organization (WHO) list of the 30 countries with the highest burden of tuberculosis and coinfection with the human immunodeficiency virus (HIV) in the world^1^. Although the country achieved a high level of treatment coverage (> 80%) in 2018, only 69.6% of new cases and 49.3% of retreatment ones were cured^2^. The target is to successfully treat 85% of all tuberculosis cases^1^.

Rio de Janeiro State (RJ) stands out for having the highest tuberculosis mortality rate in Brazil, 4.3 per 100 thousand inhabitants in 2018^2^, despite being one of its most developed states with a 0.761 HDI, the fourth highest in the country. Moreover, the municipality of Rio de Janeiro, the state capital and the second-most populous city in the country with more than 6.7 million inhabitants^3^, stands out for having an incidence of 93.7 cases per 100 thousand inhabitants, the second highest among Brazilian capitals^2^. Furthermore, 29% of all drug-resistant tuberculosis in Brazil occurs in Rio de Janeiro^4^.

Despite the Brazilian Ministry of Health suggesting the Xpert MTB RIF (Xpert) test to detect rifampicin resistance in tuberculosis cases, since 2014^5^, in Rio de Janeiro, among new cases, only 42.5% had their clinical samples evaluated by the Xpert test^2^. Among retreatment cases, only 56.8% underwent drug-susceptibility testing^2^. These tests are essential for the early diagnosis and initiation of an effective treatment for all patients with drug-resistant tuberculosis and the subsequent prevention of its transmission^6^.

This scenario of high incidence, combined with a low percentage of cure and identification of drug resistance, contributes to increasing the cases of multidrug-resistant (MDR) tuberculosis (MDR-resistance to, at least, rifampicin and isoniazid). Moreover, delays in initiating the effective treatment of MDR tuberculosis result in the continuous transmission of the disease. The condition is aggravated by its longer, more expensive, more toxic, and less effective treatment than the drug-susceptible tuberculosis one^7^.

To overcome the existing gaps in diagnosis and the rapid initiation of an effective treatment of MDR tuberculosis, we need, first, to understand the barriers imposed to the patients in this process. Thus, understanding how MDR tuberculosis is diagnosed and how its treatment affects patients is essential for the surveillance of the disease and the design of a comprehensive control program^8^.

Thus, this study aims to understand patients’ narratives about how they adjusted their practices during treatment of MDR tuberculosis, assess the course they took to diagnosis, and identify the barriers to the diagnosis and treatment of MDR tuberculosis and their consequences in Rio de Janeiro State.

## METHOD

This is a qualitative, cross-sectional and descriptive study with a non-probabilistic sampling. We applied The Consolidated Criteria for Qualitative Research Reports to guarantee the rigor of our study^9^.

In composing our patient sample, the criteria of relevance and sufficiency were respected to control for possible selection and/or confirmation biases^10^. Regarding the criterion of relevance, all patients referred to us after their medical consultations who were aged 18 years or older and under treatment for MDR tuberculosis or other mycobacterioses at an outpatient referral clinic (ORC) in Rio de Janeiro State were included. A patient with cognitive impairment, for whom the research instrument was unviable for the production of data, was excluded.

As for the sufficiency criterion, the sociodemographic profile of the patients was considered. Although the theoretical saturation criterion was applied to end the interviews, we decided to control them by selection bias, so as to achieve discursive diversity and provide opportunities for different voices to participate, based on the characteristics of the studied group (Table).

**Table:** Clinical and demographic characteristics of 31 interviewed patients.

Characteristics	Total patients in treatment (%) n = 72	Patients included in the study (%) n = 31
Sex		
Female	30 (41.7)	15 (48.4)
Male	42 (58.3)	16 (51.6)
Age^a^	35 [25–49]	48 [27–56]
Age group		
18–40	44 (61.1)	13 (41.9)
41–60	21 (29.2)	15 (48.4)
> 60	7 (9.7)	3 (9.7)
Marital status		
Married		10 (32.3)
Single		12 (38.7)
Separate		7 (22.6)
Widow(er)		2 (6.4)
Years of study		
None	3 (4.2)	2 (6.4)
1–3	4 (5.6)	4 (12.9)
4–7	26 (36.1)	7 (22.6)
8–11	25 (34.7)	14 (45.2)
≥ 12	9 (12.5)	4 (12.9)
Without information	5 (6.9)	0
Ethnicity		
Caucasian	24 (33.3)	13 (41.9)
Afro-Brazilian	48 (66.7)	18 (58.1)
Employment status		
Unemployed		13 (43.3)
Social security		12 (40.0)
Self-employed		3 (10.0)
Retired		2 (6.7)
No. of people in the household		
1		9 (29.0)
2–5		18 (58.0)
6–9		4 (13.0)
HIV status		
Negative	53 (73.6)	25 (80.6)
Positive	9 (12.5)	2 (6.4)
Unknown	10 (13.9)	4 (13.0)
Drug resistance type		
Acquired		11 (35.5)
Primary		20 (64.5)
Other factors		
Alcohol abuse		11 (35.5)
Smoking		16 (51.6)
Drug use		6 (19.3)
Diabetes mellitus		7 (22.6)

HIV: human immunodeficiency virus.

^a^ Median (interquartile range [IQR] 25%–75%).

**Figure 1:**
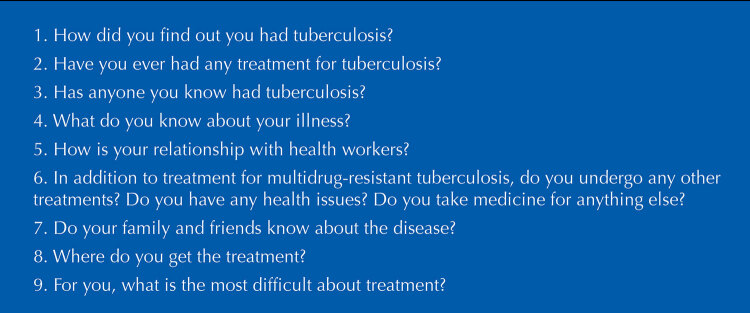
Interview guide.

Our field research was conducted from August to December 2019. The researcher visited the ORC on alternate days to avoid the concentration of participants treated by the same doctor. As for the procedures for producing data, a meeting was scheduled to present the research to the ORC health team. It was previously agreed that, when going to a medical appointment, patients would be informed by their doctor about our study. After their appointments, the patients who were interested in participating were referred to a reserved office, in which they were shown the research and the informed consent form. Semi-structured interviews, lasting approximately one hour, were conducted via a previously tested script (our interview instrument), a digital recorder, and a field diary. The script was composed of questions aimed at characterizing the subjects and contextualizing their history with tuberculosis, their knowledge about the disease, and the experiences they lived during its diagnosis and treatment (Figure 1).

To end the interviews, theoretical saturation was used as a criterion to stop us from making new interviews. Data collection was considered saturated when no new element was found, and the addition of new information failed to change our understanding of the studied phenomenon^13^. During the field research, after conducting 31 interviews, no new thematic categories relevant to the scope of our study were found. The sufficiency criterion was also considered in estimating the total sample^10,12^, in which the chance of including participants was considered based on their sociodemographic profile and on variables that would bring possible implications for the analysis of our results, i.e., gender, age group, and educational attainment^14^.

Appointments were routinely observed for aspects related to their structure and process. The outpatient clinic showed characteristics linked to a structure geared toward health treatment, aiming at the safety of its health team and patients, quality of care, and the guarantee of the functionality of the work: scheduling appointments, available staff, and reserved rooms. As for treatment, teams had doctors, nurses, and social service workers who sought support to help meet patients’ needs so treatment could have follow-ups.

Our analysis was conducted according to Bardin’s thematic content analysis^15^. The stages chosen were pre-analysis, exploration, data analysis, and interpretation. The software NVIVO version 12 was used to code the themes.

Due to the nature of the dependent variables, number (frequency) and medians (interquartile range [IQR] 25%–75%) were used to describe patients’ characteristics. For the stages of analysis and interpretation of the results of the narratives^12,16^, a theoretical framework of reference was used, in which the relevance of starting from the consonant and dissonant trajectories patients experienced was considered to obtain the correct diagnosis of MDR tuberculosis.

To classify cases into primary or acquired resistance, previous treatments were checked in the *Sistema de Informação de Tratamentos Especiais da Tuberculose* (Site TB - Special Tuberculosis Treatment Information System) and *the Sistema de Informação de Agravos de Notificação* (Sinan - Notifiable Diseases Information System). Participants had an identification number and, to protect their confidentiality, only one investigator had access to the identified codes, and they prepared the anonymous database used in this study.

### Ethical Considerations

The study protocol was approved by the research ethics committee of the Escola Nacional de Saúde Pública Sérgio Arouca - Fundação Oswaldo Cruz (CAAE 10126919.2.3001.5240).

## RESULTS

### Characteristics of the Participants

In total, 72 patients were in treatment for MDR tuberculosis at the ORC within the period of this study. Overall, we invited 32 of them; of these, 31 (97%) agreed to participate in the interviews, and we excluded one (3%) due to their cognitive limitations. Our sample had 16 (51.6%) males aged from 18 to 65 years old, with a median of 48 [27–56] years old. Regarding ethnicity, 18 (58.1%) declared themselves brown or black. Patients were at different stages of treatment, varying from the 1st to the 21st month of treatment.

Regarding marital status, 12 (38.7%) were single, and 15 (48.4%) lived with up to four people in the same household. In total, nine (29%) patients lived alone, 14 (45.2%) had between 8 and 11 years of schooling, and two, none.

As for employment status, 13 (43.3%) patients were unemployed, and 12 (40%) received some government social benefit. The average household monthly income ranged from USD 150 to USD 1,750 (median USD 317, standard deviation USD 329), and one interviewee had no income. We collected monetary values in Brazilian real (BRL) and converted them to 2019 United States dollar (USD) (BRL 4 = USD 1)^17^.

In total, four (13%) patients worked in health care services (as either pharmacists, hospital assistants or hospital laundry assistants), and nine (29%) reported previous cases of active tuberculosis among household contacts.

Regarding clinical aspects, all cases were pulmonary, two (6.4%) patients had HIV coinfection, and seven (22.6%), diabetes mellitus. As for other comorbidities, 16 (51.6%) reported a history of smoking and 11 (35.5%), of alcohol abuse. Finally, we found a high proportion of primary resistance in our group, 20 (64.5%) (Table).

### Thematic Analysis

**Figure 2:**
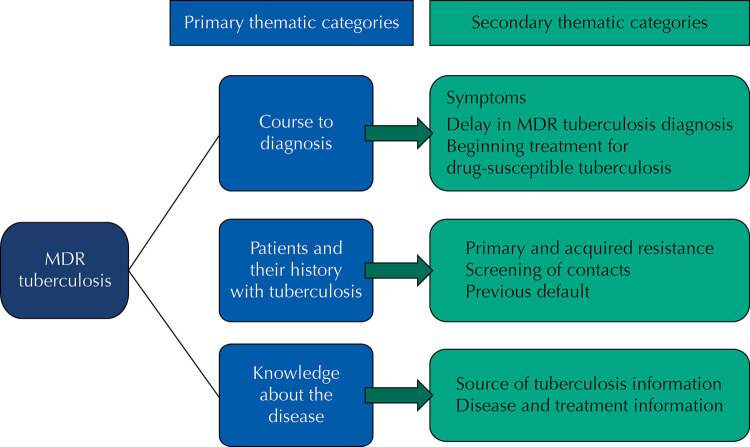
Primary and secondary thematic categories.

From the framework of thematic analysis, we used three categories: participants’ course to diagnosis, their previous history with tuberculosis, and their knowledge about the disease (Figure 2).

MDR: multidrug-resistant

#### 1) Course to diagnosis

This thematic category encompassed reports on participants’ symptoms and various mishaps experienced until diagnosis. The main problems pointed out were wrong initial diagnoses (i.e., of another disease) and failure to detect MDR tuberculosis, which led patients to undergo treatment with a standard drug-susceptible tuberculosis regimen for several months before starting an effective treatment.

*“Look, the worst part was the delay for the right diagnosis. I think that it could be improved. Like, an immediate diagnosis, correct diagnosis. I think I wouldn’t have to spend so much time... I think I would’ve been at the end of treatment. I’m practically crawling again.” (PAC 4)*

The most common symptoms reported were cough (51.6%), weight loss (41.9%), tiredness or weakness (38.7%), and fever (35.5%). In total, seven patients (22.6%) mentioned an incorrect diagnosis at their first visit to primary care: influenza, cold, pneumonia, or gastric ulcer.

In general, 16 patients (51.6%) reported initiating an incorrect treatment with a drug-susceptible tuberculosis regimen, 12 (64.5%), with a primary drug resistance one, and four (36.4%), with an acquired drug resistance one. Moreover, among them, 12 patients underwent the standard tuberculosis regimen for 6 months or more until a new diagnosis identified a therapeutic failure.

*“Then I was diagnosed with this problem. Then, I started taking medicine there. After six months, they realized that it had no effect, so I ended up here [ORC]. Here I am taking another type of medicine. Then, we’ll see…” (PAC 20)*

Even patients with previous tuberculosis treatments experienced a delay in initiating an effective one. One patient undergoing their third tuberculosis treatment had already suffered from drug resistance since their second treatment. Even so, healthcare providers prescribed a drug-susceptible tuberculosis treatment.

*“During my second treatment, the hospital professionals said that they should have already sent me here [ORC] before doing the same treatment since I had done the same treatment as the first one [for drug-sensitive tuberculosis]. They told me there that I should be here already, but they didn’t send me”. (PAC 7)*

A patient, even reporting that her mother died of MDR tuberculosis, received treatment for drug-susceptible tuberculosis without performing a drug-susceptibility testing. After 6 months, the treatment was a failure.

*“I reported everything. Finally, to sum up, after beginning six more months of treatment, I said: “This is wrong. My husband went to the administration; he started to look for information to try to get me out of there because they were not solving my problem, so…” (PAC 4)*

In total, seven patients received a MDR tuberculosis diagnosis in a public emergency department, one, in the private sector, and one, in a public research center (Fiocruz) after several visits to private doctors.

#### 2) Patients and their history with tuberculosis

In our sample, 20 (64.5%) patients mentioned primary drug resistance. Among them, 14 (70%) reported knowing someone from their family or work who had already had tuberculosis, and four were household contacts.

*“I took care of my mother, but I didn’t know that my mother had tuberculosis. My mother had pneumonia, do you understand? But then the doctor said it was that [tuberculosis]. I was taking care of her, and I wasn’t going to throw my mom out, right? Then, after my mother died, I started to feel sick”. (PAC 27)*

Patients who previously were household contacts of active tuberculosis cases mention no active case-finding. One of them, whose mother died of tuberculosis, said that she went to a primary health care unit and, even so, healthcare providers doubted that it could be tuberculosis.

*“I went to see my community health agent and my nurse, and said: “Look, I have tuberculosis” They said: “you are crazy; there is no way you can have this.” Then, I said: “I do, because of this, this, and this is happening, and it is tuberculosis.” Then they examined the symptoms, right? After that, they sent me to do the exam. Sputum is the name. It really was tuberculosis.” (PAC 26)*

Among 11 participants with acquired drug resistance, three abandoned previous treatment due to its large number of pills and injectable drugs or because they claimed they felt better and needed to go back to work.

*“I did the treatment for three or four months. I gave up. Ah, it’s very annoying, twenty big pills. About twenty, all big, in the throat, too much retching. Sometimes I choked, right?” (PAC 5)*

Regarding current treatment, 21 (68%) had had injectable drugs applied three times a week at a primary health care unit. After the end of the intensive phase, patients received self-administered medications weekly. Six patients (19%) underwent supervised treatment at a primary health care unit, two (6.5%), at home, and in two (6.5%) cases, health agents left the medication at patients’ houses once a week.

Overall, 14 (70%) patients reported that they were hospitalized during treatment due to the severity of their clinical condition.

#### 3) Knowledge about the disease

In total, 13 patients (41.9%) answered that tuberculosis was a disease transmitted by air or cough, five (16%) said that the MDR tuberculosis treatment was more difficult than the drug-susceptible tuberculosis one, and only two stated that tuberculosis could lead to death.

*“It’s worse. Tuberculosis is worse than cancer because tuberculosis comes from sneezing, coughing. If you have contact with a child, it rapidly transmits, but cancer does not transmit. Cancer is a terrible virus, but some develop it, others don’t. Tuberculosis is worse. I mean, I don’t like to talk about it to anyone.” (PAC 21)*

Another interviewee replied that it was an improperly recovered cold.

*“So, they [health workers] didn’t say much, only that I have to treat it, right? I think you have to be very careful. Many people think it is cold. They told me it is a not properly recovered cold. Is it?” (PAC 16)*

Only one patient with higher educational attainment provided a complete answer about the disease and drug resistance.

*“I read a lot of articles, right? What do I know? I know it’s a mycobacterium, right? Which can affect several organs, and which is extrapulmonary, right? You can’t stop the treatment, right? Otherwise, you produce resistance.” (PAC 29)*

## DISCUSSION

This qualitative study explores the barriers to the diagnosis and treatment of MDR tuberculosis. Our main findings are: a) participants show a high proportion of primary resistance; b) patients experience delays in the diagnosis and treatment of MDR tuberculosis; c) healthcare providers fail to value or seek the diagnosis of drug-resistant tuberculosis, thus beginning the inadequate treatment for drug-susceptible tuberculosis; d) primary health units show low report rates of active case-finding and contact monitoring; and e) patients show poor knowledge about the disease.

We were unconcerned with quantifying the delay in diagnosis, but we could observe, via the narratives, that most patients reported journeys with several comings and goings to primary health units until the beginning of the correct treatment, as described earlier. A study conducted in Rio de Janeiro showed that 79% of the interviewed patients with pulmonary tuberculosis had between two and five medical appointments before receiving the correct tuberculosis diagnosis. The median between symptom onset and diagnosis was 68 days^18^.

Overall, seven patients reported having undergone treatment for other diseases before receiving the correct MDR tuberculosis diagnosis. This initial diagnosis of other diseases may be due to nonspecific symptoms at the time of assessment which could have suggested other conditions at first^19^.

In countries with a low tuberculosis burden, even if patients show classic tuberculosis symptoms, healthcare providers often fail to test for the disease at patients’ first visits^20^. In countries with a high tuberculosis burden, such as Brazil, healthcare teams delaying the tuberculosis diagnosis may reflect poor knowledge of the disease, precarious technical performance, and/or the absence of effective diagnostic tools and follow-up routines, as tuberculosis program managers highlight^21^. A correct diagnosis requires good training and available diagnostic resources. Once the specific diagnosis is available to the health team, tuberculosis treatment should usually start.

Identifying the sources of this delay is a critical issue for effective MDR tuberculosis control. The delay in diagnosing MDR tuberculosis is associated with a critical clinical presentation, in which diagnosis follows hospitalization due to the severity of the clinical condition, as we observed in seven patients in our study, and others described^22^.

Treatment failures are the main hypothesis for the increase in drug-resistant cases but reports show the importance of primary transmission of MDR tuberculosis in recent years^22^. The high proportion (64%) of primary MDR tuberculosis we found in our study corroborates this evidence. Even though Brazil is excluded as a high-burden MDR tuberculosis country, a study conducted in Rio de Janeiro State with extensively drug resistant tuberculosis patients reported that, between 2000 and 2016, 29,3% of patients were unable to report previous treatment for drug-resistant tuberculosis, suggesting the possibility of primary transmission^26^.

Despite the Ministry of Health recommending, since 2014, the use of Xpert as an initial diagnostic test to expedite the initiation of treatment and the detection of rifampicin resistance^27^, only 45% of patients who started tuberculosis treatment in 2019 in Rio de Janeiro had an Xpert laboratory confirmation^2^. The low coverage of Xpert use among patients diagnosed with tuberculosis may relate to the decision of local program managers to not maintain their equipment (since the federal government does not cover it) or the absence of effective plans to implement the use of Xpert due to three crucial areas: a) pre-analysis (adequate sample collection and transport to the laboratory), b) analysis (quality management in carrying out tests at laboratories), and c) post-analysis (release of results, data insertion in the laboratory system, and flow in obtaining the results by the health team)^28^.

Although the WHO recommends a ≥ 2-year monitoring of MDR tuberculosis contacts for the development of active tuberculosis, despite prophylaxis^29^, none of these patients received a diagnosis by active contact case-finding. A study comparing the prevalence of tuberculin skin test positivity in contacts with MDR- and drug-susceptible tuberculosis patients found that MDR tuberculosis contacts are twice as likely (OR = 2.0; 95%CI: 1, 3–3,2) to show a positive tuberculin test. Such results suggest the importance of at least examining household contacts if index cases show MDR tuberculosis^30^. Another study, conducted in high tuberculosis-burden countries, found that 12% of new tuberculosis cases were among household contacts of MDR tuberculosis cases. In Brazil, one of the countries enrolled in this research, the percentage was 17.6%^31^.

In this study, we observed participants’ poor knowledge about tuberculosis, especially regarding its symptoms, mode of transmission, and control. Other studies also report insufficient tuberculosis knowledge in the lay population^32^ and its association with delays in tuberculosis diagnosis and treatment^33^. For individuals, in addition to knowledge about the disease, important determinants, such as demographic factors, behaviors, beliefs, perceived barriers, skills, gender, education level, and socioeconomic status are associated with patients’ delay in seeking health care^34^.

Poor knowledge about tuberculosis or misunderstandings about its transmission increase individuals’ vulnerability to it. In this scenario, we need to promote communication for the lay population in an attempt to reduce their susceptibility to health problems and adverse circumstances^35^. Tuberculosis programs should invest in broader awareness campaigns, going beyond limited educational practices, adding the distribution of pamphlets, and the setting up of posters and sporadic lectures^36^.

Another important point is the continuous training of health teams to adopt the most appropriate triage for the diagnosis of MDR tuberculosis. Gaps in healthcare providers’ knowledge about the diagnosis and care of tuberculosis indicate that their continuous training, conducted by medical/nursing schools in collaboration with tuberculosis programs, is crucial to ensure that patients are adequately screened and diagnosed, and receive the correct treatment for tuberculosis, as the End TB Strategy highlights.

One of the limitations of this study is that we only interviewed patients undergoing treatment and, therefore, were unable to collect the opinions of those outside that service. Although our sample is unable to represent the barriers faced by all patients diagnosed and treated in Rio de Janeiro State, it may suggest a starting point for understanding the barriers patients face in accessing the diagnosis and effective treatment of MDR tuberculosis.

## FINAL REMARKS

For the diagnosis of active tuberculosis, the public health system offers rapid molecular testing, sputum smear microscopy, culture for mycobacteria, and drug-susceptibility testing. Although the Xpert MTB/RIF molecular test has been available in the Brazilian public health system since 2014 for the diagnosis of active tuberculosis, we found that delays in the diagnosis of MDR tuberculosis was a factor that stood out in patients’ narratives.

The delay in the diagnosis of MDR tuberculosis leads patients to face unnecessary drug regimens which worsen their clinical condition and maintain the chain of transmission of the disease. Although Rio de Janeiro State has a laboratory network capable of diagnosing MDR tuberculosis, this has been insufficient to speed up the beginning of effective treatment. We need to improve referral systems, and access to early diagnosis and treatment; conduct an active search for contacts; intensify the training of healthcare providers, in collaboration with medical and nursing schools, and public and private systems; and promote campaigns on the signs and symptoms of tuberculosis for the lay population.
